# Mesenchymal stem cells improve cardiac function after myocardial infarction in rats without long-term survival: a serial 7.0T MRI study

**DOI:** 10.1186/1532-429X-17-S1-P132

**Published:** 2015-02-03

**Authors:** Xiuyu Chen

**Affiliations:** Radiology, State Key Laboratory of Cardiovascular Disease, Fuwai Hospital, National Center for Cardiovascular Diseases, Chinese Academy of Medical Sciences and Peking Union Medical College, Beijing, People’s Republic of China, Beijing, China

## Background

Bone marrow derivedmesenchymal stem cells (MSCs) are a promising approach to the treatment of cardiac injury after myocardial infarction (MI). However, little is known about the underlying mechanisms and the fate of the transplanted cells.Our aim was to in vivo monitoring the magnetically labeled MSCs after transplantation into infarcted rat hearts and determining the effects on cardiac function using a 7.0 T magnetic resonance imaging (MRI) scanner.

## Methods

Rat MSCs (male) were dual-labeled with fluorescent micron-sized particles of iron oxide (MPIO) and CM-DiI. Seven days after MI, rats (syngeneic females) were randomized to injections of labeled MSCs (2×10^6^ cells/50μL) or saline (50μL) into the border zone of infarcted myocardium. MRI was used to evaluate stem cell migration, signal intensity changes and cardiac function at baseline (1 day before transplantation), 3 days, 2 weeks and 4 weeks after transplantation, respectively. At each time point after MRI examination, myocardial tissue from 6~8 hearts was analyzed by postmortem analyses.

## Results

MR hypointensities caused by the MPIOs were detected on T2*-weighted imaging at all times after MSCs transplantation. As time progressed, the signal gradually weakened and the area shrank. Serial cine-MRI showed that MSCs transplantation attenuated progressive left ventricular dilatation and dysfunction compared with controls at 4 weeks. By real-time polymerase chain reaction with Y-chromosome specific primers, the number of grafted MSCs in the hearts decreased rapidly from 11.5% (3 days) to ~0.1% (4 weeks). Double staining for iron and CD68 (resident macrophage marker) showed that most of the iron-positive cells were cardiac macrophages at 4 weeks. In addition, MSC-treated hearts had significantly increased capillary density in the peri-infarct region, and lower cardiomyocytes apoptosis and collagen deposition.

## Conclusions

The survival of injected MPIO-labeled MSCs is poor at 4 weeks after transplantation, and the MR hypointensities mainly arise from cardiac macrophage that engulfed the MPIO particles. However, MSCs attenuate left ventricular dilatation and dysfunction after MI, which may attribute to enhanced angiogenesis, inhibition of host cell apoptosis and fibrosis.

## Funding

This study was supported by a research grant of National Natural Science Foundation of China (81130029).

